# Disseminated Tuberculosis With Ocular Involvement After 12 Years of Anti–TNF‐*α* Therapy

**DOI:** 10.1155/crop/1351144

**Published:** 2026-10-18

**Authors:** Muhammad Ali Muslimani, Alberto Ardizzone, Silvia Rossi, Francesco Siviero, Armanda Leka, Marco Vecchia, Alessandra Ricciardi, Pietro Valsecchi, Raffaele Bruno, Angela Maria Di Matteo

**Affiliations:** ^1^ Department of Clinical Surgical Diagnostic and Pediatric Sciences University of Pavia Pavia Italy unipv.eu; ^2^ Division of Infectious and Tropical Diseases IRCCS S. Matteo Hospital Foundation Pavia Italy; ^3^ Division of Ophthalmology IRCCS S. Matteo Hospital Foundation Pavia Italy; ^4^ Division of Rheumatology IRCCS S. Matteo Hospital Foundation Pavia Italy

**Keywords:** adalimumab, anti–TNF-*α* therapy, choroidal granuloma, disseminated tuberculosis, infectious diseases, *Mycobacterium tuberculosis*, ocular tuberculosis, ophthalmology, rheumatoid arthritis, rheumatology

## Abstract

**Background:**

Ocular tuberculosis (TB) is an uncommon yet potentially vision‐threatening manifestation of *Mycobacterium tuberculosis*, particularly difficult to diagnose in immunocompromised individuals. The increasing use of anti–tumor necrosis factor‐*α* (anti–TNF‐*α*) agents has markedly heightened the risk of TB reactivation, often with disseminated or extrapulmonary involvement despite negative baseline screening.

**Case Presentation:**

We describe a 52‐year‐old woman with rheumatoid arthritis receiving long‐term adalimumab and methotrexate who developed disseminated TB with pulmonary, splenic, and ocular involvement after 12 years of anti–TNF‐*α* therapy. Baseline screening had shown a negative tuberculin skin test and an indeterminate interferon‐*γ* release assay. She presented with constitutional and respiratory symptoms, whereas chest computed tomography demonstrated diffuse micronodular infiltrates consistent with miliary TB. Microbiological confirmation was obtained via bronchoalveolar lavage. Ophthalmologic evaluation revealed bilateral choroidal granulomas with subretinal edema, leading to a diagnosis of ocular TB. Standard antitubercular therapy (ATT) was initiated but required modification due to drug‐induced liver injury. Adjunctive systemic corticosteroids were introduced after antimicrobial therapy and gradually tapered. The patient achieved progressive clinical, radiological, and ophthalmologic improvement, with gradual regression of choroidal lesions.

**Discussion:**

This case underscores the persistent long‐term risk of TB reactivation during prolonged anti–TNF‐*α* therapy and highlights the limitations of current screening strategies in immunosuppressed patients. Ocular TB requires integration of systemic, clinical, and imaging findings; in this patient, microbiologically confirmed disseminated TB together with characteristic bilateral choroidal granulomas supported a definitive diagnosis. Management relies on prompt ATT, discontinuation of immunosuppression, and judicious use of adjunctive corticosteroids. Long‐term follow‐up is essential given the risk of persistent lesions and late recurrence. Reintroduction of biologic therapy may be feasible in selected cases but requires careful risk stratification.

**Conclusion:**

TB should remain a diagnostic consideration in patients receiving biologic therapy who develop compatible systemic or ocular manifestations, even after long‐term treatment and negative baseline screening. Close multidisciplinary collaboration is essential for the diagnosis and management of ocular TB in this population.

Learning Points

1. Tuberculosis (TB) reactivation can occur during anti–tumor necrosis factor‐*α* (anti–TNF‐*α*) therapy even after prolonged treatment duration and negative baseline screening.
2. Ocular TB requires an integrative diagnosis based on clinical findings, imaging, and evidence of systemic TB; in the presence of characteristic ocular lesions and microbiologically confirmed disseminated TB, a definitive diagnosis is warranted.
3. Interferon‐*γ* release assays and tuberculin skin tests (TSTs) may yield false‐negative or indeterminate results in immunosuppressed patients.
4. Ocular involvement may be part of systemic disseminated TB, even in the absence of overt ocular symptoms.
5. Prompt discontinuation of immunosuppressive therapy and initiation of a full course of antitubercular therapy (ATT) are essential in ocular TB associated with active systemic disease.
6. Adjunctive corticosteroids may be useful in selected cases of ocular inflammation but should always be administered in conjunction with effective antimycobacterial therapy.
7. Anti–TNF‐*α* therapy may be associated with a persistent long‐term risk of TB reactivation beyond the early treatment period.
8. Long‐term follow‐up is critical due to the risk of persistent lesions, delayed resolution, and disease relapse.
9. Reintroduction of biologic therapy is feasible but requires careful risk stratification, with consideration of non‐TNF–targeting alternatives in high‐risk patients.
10. Multidisciplinary management (infectious disease, ophthalmology, and rheumatology) is essential for optimal diagnosis, treatment, and follow‐up.

## 1. Introduction

Ocular TB is a clinical entity caused by or associated with *Mycobacterium tuberculosis*, capable of involving virtually any ocular structure and presenting with a broad spectrum of manifestations. It is classified as a form of extrapulmonary TB and is of particular clinical relevance in immunocompromised individuals, in whom delayed recognition may result in irreversible visual impairment and systemic complications [1].

Ocular TB accounts for a small proportion of TB cases worldwide, with an estimated prevalence of less than 6% in Italy, and is likely underdiagnosed due to the absence of standardized diagnostic criteria and its heterogeneous clinical presentation [2]. Its true burden remains difficult to ascertain, reflecting both diagnostic uncertainty and variability in clinical suspicion [3].

The increasing use of biologic agents, particularly anti–TNF‐*α* therapies, has significantly altered the epidemiology of TB. By impairing granuloma formation and maintenance, these agents predispose to reactivation of latent tuberculosis infection (LTBI) and facilitate hematogenous dissemination, often resulting in extrapulmonary or atypical disease presentations. Notably, TB may develop despite negative baseline screening, and the duration of susceptibility during long‐term biologic therapy remains incompletely defined. Although most cases of anti‐TNF–associated TB occur during the first year of treatment [4, 5], delayed presentations after many years of continuous therapy have occasionally been reported, highlighting the need for lifelong clinical vigilance.

We report a case of disseminated TB with pulmonary, splenic, and ocular involvement occurring after 12 years of anti–TNF‐*α* therapy in a patient with rheumatoid arthritis (RA), despite negative baseline screening and persistently indeterminate immunologic testing. This case, developing after 12 years of uninterrupted adalimumab treatment, represents one of the longest reported intervals between initiation of anti–TNF‐*α* treatment and development of disseminated ocular TB. It highlights the limitations of current screening strategies, the potential for very late reactivation during prolonged biologic exposure, and the diagnostic and therapeutic challenges of ocular TB in immunosuppressed patients. It further underscores the importance of close collaboration among infectious disease specialists, ophthalmologists, and rheumatologists in optimizing patient management and outcomes.

## 2. Case Presentation

A 52‐year‐old Italian woman was receiving immunosuppressive therapy with the anti–TNF‐*α* agent adalimumab and methotrexate for seronegative, nonerosive RA. Pretreatment screening for chronic and opportunistic infections included a negative TST and a single QuantiFERON‐TB Gold Plus (QFT‐GP) assay with an indeterminate result. Chest radiography showed no evidence of previous or active TB. Serological testing revealed a positive anti‐HBsAg titer and no evidence of exposure to hepatitis C virus (HCV).

Approximately 12 years later, the patient was admitted to our hospital with a 2‐month history of productive cough associated with constitutional symptoms, including evening fever, night sweats, and unintentional weight loss. Laboratory investigations revealed elevated C‐reactive protein (CRP), mild anemia, lymphopenia, and slightly elevated lactate dehydrogenase (LDH). Contrast‐enhanced chest computed tomography (CT) demonstrated bilateral diffuse micronodular consolidations with perihilar and mediastinal lymphadenopathy (Figure 1). Serum angiotensin‐converting enzyme (ACE) and lysozyme levels were within normal limits. A repeat QFT‐GP assay yielded an indeterminate result. Active pulmonary TB was therefore suspected; adalimumab and methotrexate were immediately discontinued, and the patient was referred to the Infectious Diseases Department.

**Figure 1:**
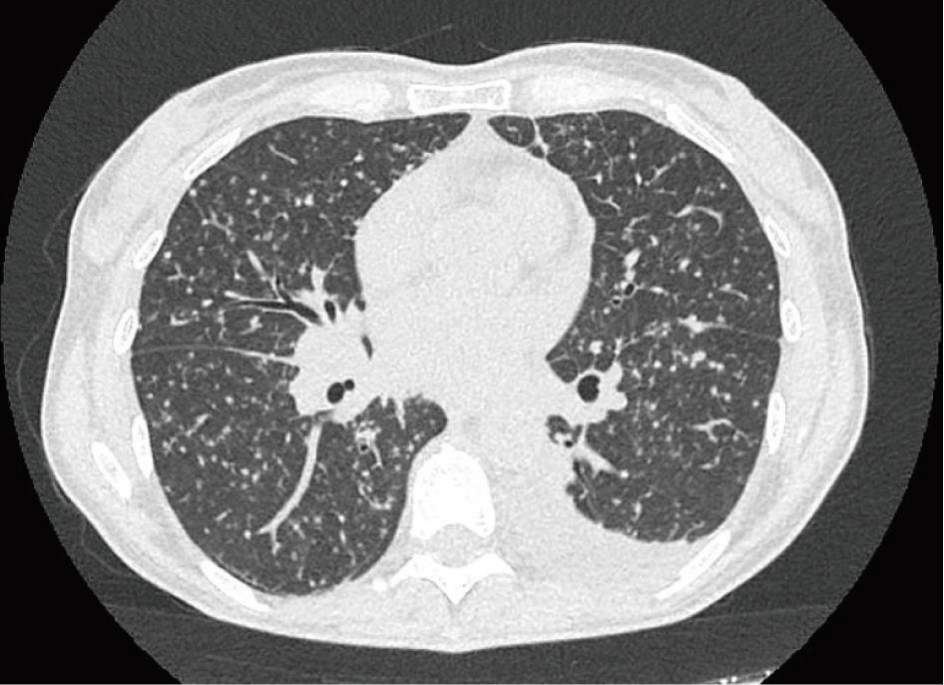
Bilateral pulmonary consolidations with a diffuse micronodular pattern on CT scan.

Given the patient′s relative clinical stability, antimicrobial therapy was withheld until bronchoalveolar lavage (BAL) could be performed. Bronchoscopy revealed diffuse millimetric whitish nodules involving both the tracheal and bronchial mucosa. Microscopy and microbiological analysis of BAL fluid confirmed the diagnosis of miliary pulmonary TB: Acid‐fast bacilli (AFB) were detected, and the Xpert MTB/RIF assay identified *M*. *tuberculosis* DNA without genotypic resistance to rifampicin. Notably, sputum samples were negative for AFB; therefore, the patient was not considered infectious. ATT with rifampicin, isoniazid, ethambutol, and pyrazinamide was initiated. Cultures from both sputum and BAL fluid subsequently grew pan‐susceptible *M*. *tuberculosis*. Histopathological examination of the endobronchial lesions revealed multiple areas of necrosis with granuloma formation, without detectable AFB on Ziehl–Neelsen staining.

When questioned about possible visual symptoms potentially related to ATT 3 days after its introduction, the patient reported occasional binocular floaters that had begun before hospital admission. Prompt ophthalmologic evaluation was performed. The best corrected visual acuity (BCVA) was 20/20 in the right eye and 20/25 in the left eye. Intraocular pressure was within normal limits in both eyes. Slit‐lamp examination showed no evidence of anterior chamber inflammation, and fundus examination revealed no vitreous cells or signs of vitritis. A solitary choroidal granuloma was identified in each eye (Figure 2). In the right eye, the lesion was located nasal to the optic disc in the midperipheral retina and measured approximately 3150 × 3170 *μ*m, with a maximum thickness of 853 *μ*m. In the left eye, the granuloma was located in the superotemporal macular region, involving the fovea through overlying subretinal fluid, and measured approximately 2996 × 4237 *μ*m, with a maximum thickness of 925 *μ*m. Optical coherence tomography (OCT) demonstrated a thin subretinal edema layer in both eyes (Figures 3, 4, and 5). No molecular or culture samples were obtained from the vitreous or aqueous humor. Based on the presence of characteristic bilateral choroidal granulomas in the setting of microbiologically confirmed disseminated TB, after exclusion of alternative causes, a diagnosis of ocular TB was made.

**Figure 2:**
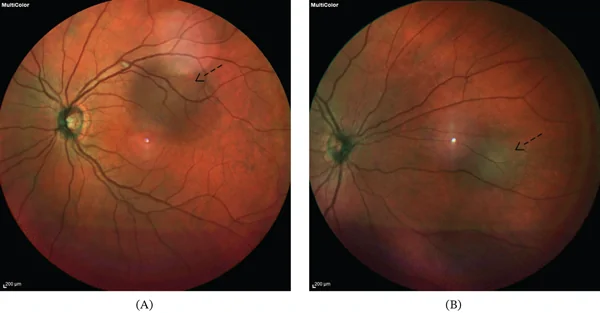
Multicolor fundus imaging revealing bilateral solitary choroidal granulomas. Arrows indicate a solitary choroidal tuberculoma. ( A) Left eye and (B) right eye.

**Figure 3:**
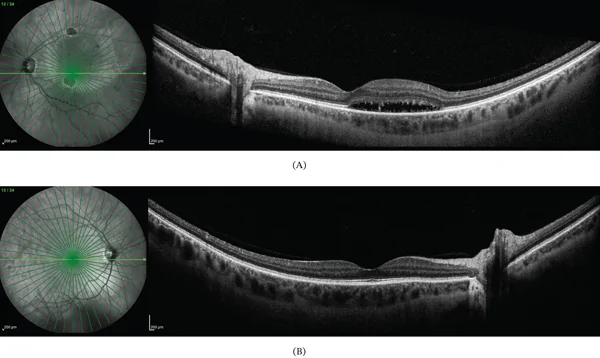
Fundus photographs and corresponding OCT images with line scans passing through the foveal center. (A) Left eye and (B) right eye.

**Figure 4:**
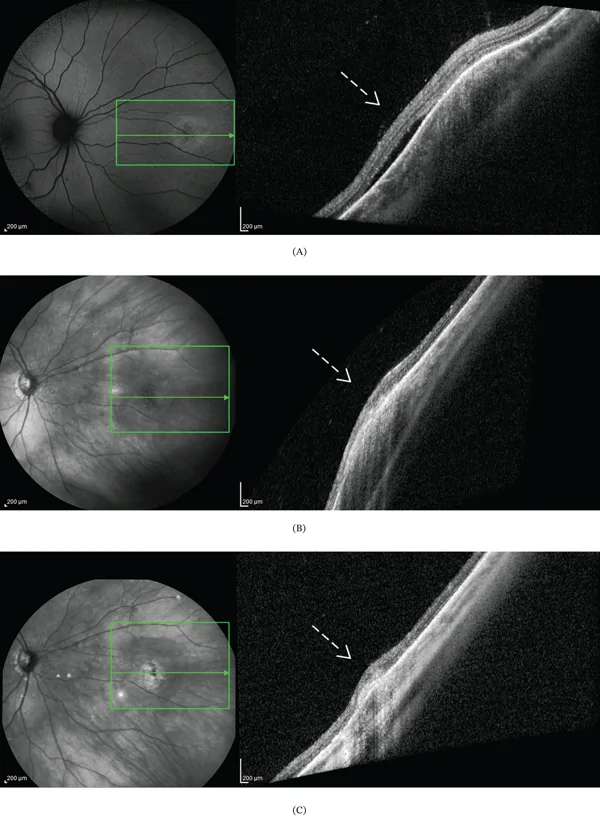
Fundus photographs and corresponding OCT images of the right eye. Arrows indicate a solitary choroidal tuberculoma. (A) Baseline, (B) at 4 months, and (C) at 12 months.

**Figure 5:**
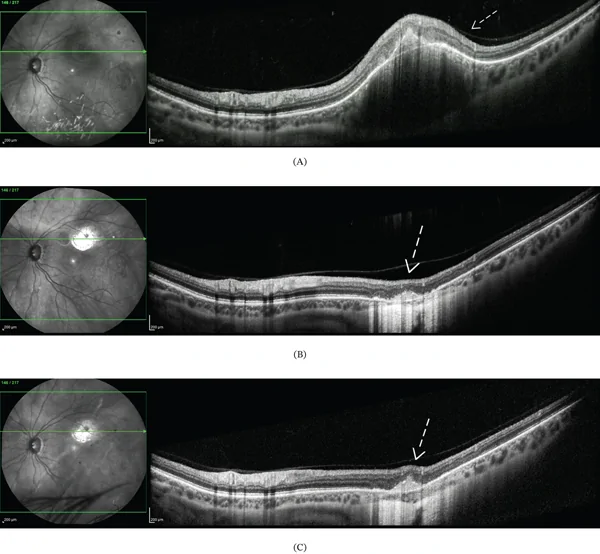
Fundus photographs and corresponding OCT images of the left eye. Arrows indicate a solitary choroidal tuberculoma. (A) Baseline, (B) at 4 months, and (C) at 12 months.

Following multidisciplinary discussion, adjunctive systemic corticosteroids were initiated at a prednisone‐equivalent dose of 0.6 mg/kg/day approximately 7 days after the start of effective ATT. Although higher initial doses of systemic corticosteroids are commonly used for severe posterior uveitis, a starting dose of 0.6 mg/kg/day was selected to balance control of the ocular inflammatory response against the risk of additional immunosuppression in the setting of active disseminated TB. The patient′s recent exposure to adalimumab was also taken into consideration in this decision. Corticosteroids were introduced only after effective antimycobacterial treatment had been established because OCT demonstrated bilateral choroidal granulomas associated with subretinal edema, raising concern for ongoing inflammatory damage despite preservation of central vision. In addition, the patient remained persistently febrile during the first week of treatment despite appropriate ATT and microbiological confirmation of drug‐susceptible TB. After exclusion of alternative causes of persistent fever, corticosteroids were administered to control the inflammatory response likely associated with disseminated TB. The patient became afebrile shortly after corticosteroid initiation. Because rifampicin increases systemic corticosteroid clearance and reduces prednisone exposure, the potential for reduced corticosteroid effect during concomitant ATT was considered; clinical response was monitored closely, and no dose escalation beyond 0.6 mg/kg/day was required.

A comprehensive diagnostic workup was performed to identify other potential foci of infection in the context of disseminated TB. Transthoracic echocardiography (TTE) revealed a minimal amount of partially organized pericardial effusion without hemodynamic compromise. Additionally, contrast‐enhanced abdominal CT showed multiple hypodense lesions in the splenic parenchyma, suggestive of miliary TB involvement (Figure 6). Contrast‐enhanced brain CT excluded cerebral localization. Subsequent lumbar puncture yielded clear cerebrospinal fluid (CSF) with normal opening pressure. CSF glucose, protein, and cell counts were within normal limits, and both molecular and culture analyses for *M*. *tuberculosis* were negative. Blood and urine cultures were also negative.

**Figure 6:**
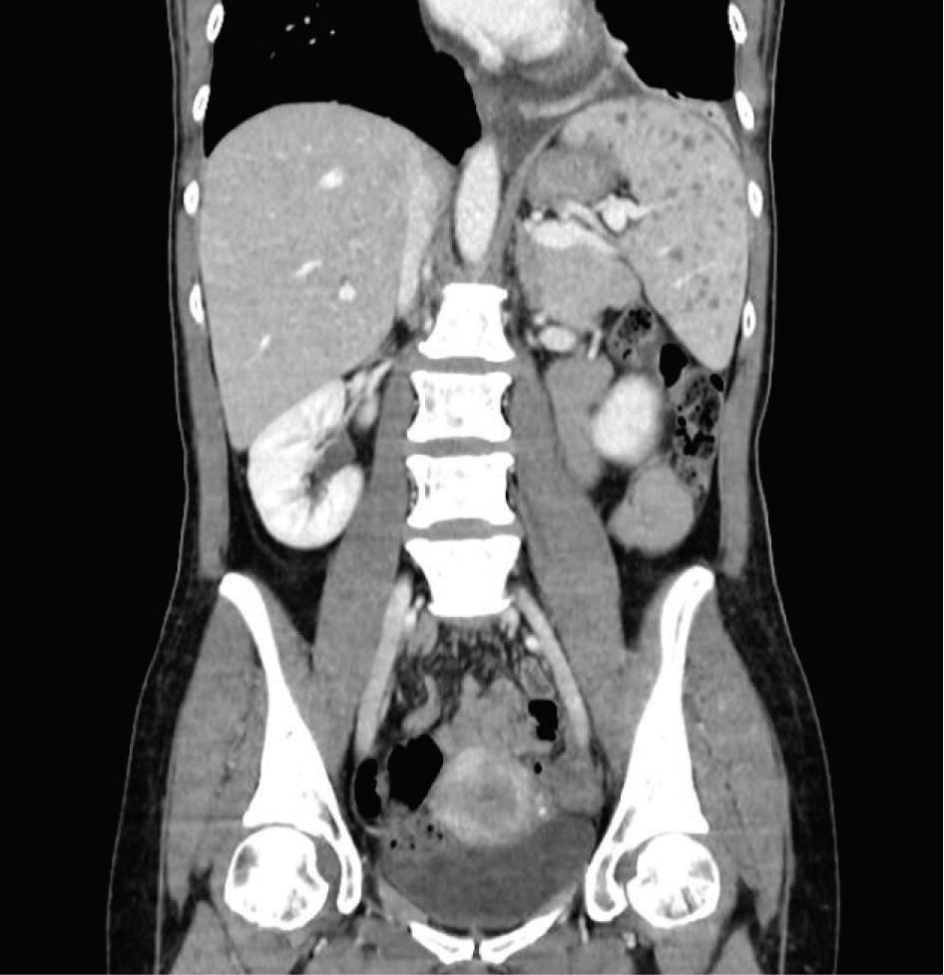
Miliary splenic tuberculosis on abdominal CT scan.

During the first week of quadruple ATT, cholestatic and hepatocellular enzyme levels progressively increased, with alanine aminotransferase (ALT) and aspartate aminotransferase (AST) reaching 327 and 393 U/L, respectively. Drug‐induced liver injury (DILI) was suspected; ATT was therefore temporarily discontinued. Systemic corticosteroid therapy, which had been initiated approximately 7 days after ATT at 0.6‐mg/kg/day prednisone equivalent, was maintained at the full dose during the DILI episode because the ocular and systemic inflammatory indications persisted. Once transaminase levels normalized, antitubercular agents were reintroduced sequentially, one drug at a time, with close biochemical monitoring. Pyrazinamide was replaced with moxifloxacin to reduce the risk of recurrent hepatotoxicity. No further episodes of hypertransaminasemia occurred.

Serial ophthalmologic evaluations demonstrated progressive improvement in visual function, although occasional floaters and episodes of metamorphopsia persisted. Visual evoked potential (VEP) testing excluded neurological or iatrogenic involvement of the retinal and postretinal visual pathways. Serial high‐resolution Heidelberg OCT enabled precise longitudinal assessment of lesion morphology (Figures 4 and 5). Lesion thickness, rather than diameter, was used to quantify disease progression because it represents a more reproducible and less operator‐dependent parameter. In the left eye, lesion thickness increased from 925 *μ*m at presentation to approximately 1300 *μ*m after 1 month, accompanied by a transient increase in subretinal fluid and overlying mixed‐reflectivity material. From the fourth month onward, OCT demonstrated progressive regression of the lesion, with a reduction in thickness, substantial resolution of subretinal fluid, and gradual organization of the overlying material. By the sixth month, the granuloma had collapsed, with evolution toward chorioretinal atrophy and retinal pigment epithelium atrophy. The right eye exhibited a similar temporal course, ultimately progressing to atrophic remodeling. Respiratory and constitutional symptoms also gradually resolved during therapy. Once clinically stable, the patient was discharged with scheduled multidisciplinary follow‐up.

Prednisone was subsequently tapered gradually over 2 months, with weekly dose reductions and progressively smaller decrements at lower doses until complete discontinuation. The taper was guided by the clinical and ophthalmologic response, with no recurrence of ocular inflammation. The ATT regimen was continued for a total of 12 months. The rifampicin‐based regimen was continued throughout treatment; after 2 months, ethambutol and moxifloxacin were discontinued, whereas isoniazid and rifampicin were continued for a further 10 months. Twelve months after the index event, isoniazid and rifampicin were discontinued. Although the ocular lesions progressively regressed, residual bilateral chorioretinal scarring secondary to the granulomas remained detectable. Nonetheless, the patient retained excellent visual acuity. During follow‐up, lengthy interruption of adalimumab and methotrexate was not associated with a clinically significant RA flare, and the patient remained in sustained remission. Consequently, neither treatment was restarted during the 12‐month ATT course.

## 3. Discussion

This case highlights three clinically salient and underrecognized challenges in the management of TB in patients undergoing prolonged anti–TNF‐*α* therapy: (i) the potential for very late TB reactivation despite negative baseline screening, (ii) the diagnostic complexity of ocular TB in immunosuppressed patients, and (iii) the need for individualized therapeutic strategies in the context of immunosuppression and drug‐related toxicity.

From a pathophysiological standpoint, ocular TB arises through a combination of direct infection, hematogenous dissemination, and immune‐mediated mechanisms. Among these, hematogenous spread from a pulmonary or extrapulmonary focus represents the predominant pathway, particularly in disseminated disease, with the uveal tract serving as the principal site of involvement due to its high vascularity and oxygen tension [6]. This mechanism is consistent with the present case, in which miliary pulmonary and splenic TB were accompanied by bilateral choroidal granulomas, supporting secondary ocular seeding. The choroid is especially susceptible to bacillary localization, and posterior segment involvement—manifesting as choroidal tubercles or tuberculomas—constitutes the most frequent ocular presentation [7–9]. These lesions may evolve with localized inflammation, subretinal edema, and retinal pigment epithelium disruption, potentially progressing to retinal detachment or subretinal abscess formation in advanced stages [10]. With timely initiation of therapy, lesions may regress or resolve with residual atrophic scarring [11]. In the present case, early recognition and treatment likely contributed to lesion stabilization and gradual regression.

The diagnosis of ocular TB remains clinically challenging because ocular involvement may mimic other inflammatory or infectious causes of uveitis and direct microbiological confirmation from ocular tissue is rarely achievable due to the paucibacillary nature of intraocular infection and the invasiveness of tissue sampling, with low sensitivity of both culture and molecular assays on ocular specimens [12, 13]. Immunologic tests, including the TST and interferon‐*γ* release assays, provide supportive but imperfect evidence; their diagnostic performance is significantly reduced in immunosuppressed individuals, in whom false‐negative or indeterminate results are common [12–14]. Consequently, diagnosis relies on a composite approach integrating compatible ocular findings, evidence of systemic TB, immunologic testing, and exclusion of alternative etiologies [9, 12, 15]. In the present case, the combination of characteristic bilateral choroidal granulomas, microbiologically confirmed disseminated TB, and exclusion of alternative causes supports a definitive diagnosis of ocular TB. This presentation is consistent with the clinical frameworks proposed by the Collaborative Ocular Tuberculosis Study (COTS) [16] and the 2022 British Thoracic Society (BTS) Clinical Statement [1]; these documents should be understood as aids to clinical assessment and treatment decision‐making rather than as rigid diagnostic criteria. Accordingly, a full course of ATT was administered.

The patient′s prolonged exposure to anti–TNF‐*α* therapy represents a central determinant of disease risk. Tumor necrosis factor‐*α* is essential for granuloma formation and maintenance of latent infection; its pharmacologic inhibition disrupts host immune containment, predisposing to reactivation and hematogenous dissemination of *M*. *tuberculosis*. This association is well established, with anti–TNF‐*α* agents significantly increasing the incidence of both pulmonary and extrapulmonary TB, often with atypical or disseminated presentations [14, 17–20]. Notably, incidence rates exceeding 1000 per 100,000 patient‐years have been reported in RA cohorts receiving anti–TNF‐*α* therapy [17, 21]. Risk is further amplified by advanced age, prior TB exposure, and concomitant immunosuppressive agents, including methotrexate and corticosteroids [18, 21]. Importantly, TB may develop despite negative baseline screening and even after appropriate prophylaxis [18, 19]. The unusually prolonged latency observed in this case highlights that the risk of reactivation is not confined to the early treatment period but may persist indefinitely, challenging the adequacy of current screening paradigms based on a single pretreatment assessment. Although most TB cases associated with anti–TNF‐*α* therapy occur within the first 6–12 months after treatment initiation, registry studies demonstrate that the risk persists throughout treatment, and very late presentations after many years of continuous biologic exposure, although rarely reported, remain biologically plausible because TNF‐*α* blockade continuously compromises granuloma integrity [22, 23]. Consequently, the 12‐year latency observed in our patient reinforces the concept that TNF‐*α* blockade confers a persistent rather than time‐limited risk of TB reactivation or newly acquired infection.

This case raises the question of whether TB screening should be repeated during prolonged biologic therapy. Current international recommendations uniformly advocate baseline screening before initiation of anti–TNF‐*α* therapy [24]. However, more recent rheumatology guidelines recommend repeat LTBI testing during treatment for patients with new TB exposure, ongoing epidemiologic risk, or residence in intermediate‐ or high‐incidence settings, whereas routine serial testing is generally not recommended in low‐incidence populations without new risk factors [25]. Importantly, routine annual testing in all patients is not universally recommended because of limited evidence and reduced performance of immunologic assays in immunosuppressed individuals. This case illustrates the limitations of relying exclusively on baseline screening and underscores the importance of continued clinical surveillance for TB throughout long‐term biologic treatment, even where routine repeat immunological testing is not mandated.

Indocyanine green angiography (ICGA) may provide additional diagnostic information in ocular TB because it can reveal subclinical choroidal lesions that are not readily apparent on fundus examination or conventional angiography. Although ICGA was not available for the present case, and therefore no ICGA images could be included, its potential role should be acknowledged, particularly in patients with multifocal or clinically subtle choroidal involvement. In this patient, the bilateral characteristic choroidal granulomas identified by fundus imaging and OCT, together with microbiologically confirmed disseminated TB, provided sufficient clinical support for the diagnosis.

Management of ocular TB in this context requires a multidisciplinary and individualized approach. Immediate discontinuation of immunosuppressive therapy, when clinically feasible, and prompt initiation of standard ATT remain the cornerstone of treatment [12, 14]. In the present case, therapy was complicated by DILI, necessitating temporary interruption and stepwise reintroduction of agents, with substitution of pyrazinamide by moxifloxacin. This underscores the importance of close biochemical monitoring and flexible regimen adjustment in complex patients.

The role of adjunctive corticosteroids in ocular TB remains controversial. Although their use is biologically plausible due to suppression of immune‐mediated inflammation, available evidence does not demonstrate a consistent benefit in visual outcomes, inflammation resolution, or relapse prevention when compared with ATT alone [26–30]. Reported rates of inflammation control and visual improvement are high with ATT alone, and comparative analyses show minimal differences with the addition of corticosteroids [28–30]. Moreover, corticosteroid use in the absence of effective antimicrobial therapy is associated with adverse outcomes and increased relapse risk [31–33]. Accordingly, current practice supports a selective, case‐by‐case approach, reserving corticosteroids for severe or sight‐threatening inflammation and always in conjunction with appropriate ATT [27]. In our patient, adjunctive systemic corticosteroids were introduced approximately 1 week after initiation of ATT because the choroidal granulomas were associated with subretinal edema, indicating active posterior segment inflammation with potential for structural damage. Persistent fever despite appropriate ATT, after exclusion of alternative causes, also suggested an ongoing inflammatory response in the setting of disseminated TB. Prednisone was initiated at 0.6 mg/kg/day. Although systemic corticosteroids are often initiated at approximately 1–2 mg/kg/day in severe posterior uveitis, a lower initial dose was selected to balance control of posterior segment inflammation against the risk of additional immunosuppression in the setting of active disseminated TB and recent adalimumab exposure [1, 34]. Because rifampicin increases systemic corticosteroid clearance and reduces prednisone exposure, corticosteroid requirements may be higher during concomitant rifampicin therapy [35]. In this patient, the clinical response was satisfactory, and no dose escalation beyond 0.6 mg/kg/day was required. Defervescence occurred shortly after corticosteroid initiation. Although this temporal association does not establish causality, it supports the rationale for adjunctive corticosteroids in carefully selected patients with both ocular inflammation and significant systemic inflammatory manifestations. Prednisone was subsequently tapered gradually over 2 months, with weekly dose reductions and progressively smaller decrements at lower doses, until complete discontinuation. The taper was guided by the clinical and ophthalmologic response, with no recurrence of ocular inflammation.

An additional therapeutic consideration in choroidal granulomas with clinically significant subretinal fluid is adjunctive intravitreal therapy. Intravitreal antivascular endothelial growth factor (anti‐VEGF), alone or combined with intravitreal moxifloxacin, has been reported as an adjunct to systemic ATT and corticosteroids, particularly when a large or fovea‐involving granuloma produces substantial subretinal fluid or threatens vision. Case reports and a recent retrospective series describe anatomical improvement with intravitreal anti‐VEGF plus moxifloxacin, although the evidence remains limited and consists predominantly of case reports and small observational series [36]. Intravitreal anti‐VEGF may be particularly relevant when subretinal fluid persists despite systemic treatment, whereas intravitreal moxifloxacin has been proposed as an adjunctive antimicrobial strategy in selected severe lesions. In the present case, however, the subretinal fluid was thin and progressively resolved with systemic ATT and corticosteroid therapy, and therefore intravitreal treatment was not clinically required. The patient′s preference to avoid invasive ocular procedures further supported the decision not to pursue intravitreal therapy.

Beyond acute management, long‐term considerations include the risk of TB relapse and the potential reintroduction of biologic therapy. Relapse rates following reinitiation of biologics are relatively low but nonnegligible, with pooled estimates of approximately 3.8% and a median time to recurrence of 8.5 months [37]. Most cases can be successfully managed without microbiological failure [38, 39]. Reintroduction is generally deferred until completion of ATT, although earlier resumption may be considered in selected cases under close surveillance [38, 39]. Non‐TNF–targeting biologic agents, including rituximab, abatacept, and tocilizumab, may offer alternative treatment options for patients requiring renewed immunosuppression after TB, although the magnitude of TB risk may vary among agents and should be considered on an individual basis [21, 40, 41].

In the present case, adalimumab and methotrexate were discontinued immediately when active disseminated TB was suspected, given the potential for continued TNF‐*α* blockade to impair granuloma maintenance and host control of infection. Both agents remained withheld throughout the 12‐month ATT course, with priority given to achieving sustained microbiological and clinical control of TB. Despite interruption of immunosuppressive therapy, the patient did not experience a clinically significant RA flare during follow‐up and remained in sustained remission. Consequently, reintroduction of methotrexate or biologic therapy was not required. This outcome reflects the individual disease course and should not be interpreted as evidence that discontinuation of adalimumab is universally unlikely to result in an RA flare within the first 12 months. Decisions regarding resumption of antirheumatic therapy should instead be individualized according to disease activity, TB treatment response, and the overall risk–benefit profile, in collaboration with rheumatology and infectious‐disease specialists. If clinically significant RA activity had emerged, management would have required reassessment of disease activity and consideration of the lowest‐risk antirheumatic strategy compatible with infection control. A non‐TNF–targeting agent could be considered if biologic therapy were subsequently necessary, with the timing of reintroduction determined through a carefully justified, multidisciplinary plan.

Long‐term follow‐up is essential, particularly in patients with prior immunosuppressive exposure. Although most patients demonstrate clinical improvement within the first months of therapy, residual lesions and late recurrences are well documented [12, 27, 42, 43]. Ophthalmologic monitoring is critical to assess treatment response, guide corticosteroid tapering, and detect complications. Follow‐up for at least 12 months is generally recommended, with extended surveillance up to 24 months or longer in high‐risk patients, given that relapse may occur beyond the first year and is often associated with suboptimal initial response [44–47]. The persistence of choroidal lesions in our patient at follow‐up, albeit reduced in size, reinforces the need for protracted monitoring.

## 4. Conclusion

This case underscores that TB in patients receiving anti–TNF‐*α* therapy may manifest as late‐onset, disseminated, and diagnostically elusive disease, even after years of treatment and in the setting of inconclusive immunologic screening. It highlights the inherent limitations of current screening strategies and reinforces the need to maintain a high index of suspicion for TB in immunocompromised individuals throughout the entire course of biologic therapy and to consider repeat LTBI assessment in patients with ongoing epidemiologic exposure or other high‐risk circumstances. In the present patient, characteristic bilateral choroidal granulomas in the setting of microbiologically confirmed disseminated TB supported a definitive diagnosis of ocular TB and warranted a full 12‐month course of ATT. Optimal management requires early recognition, prompt initiation of appropriate antimycobacterial therapy, and coordinated multidisciplinary care involving infectious disease specialists, ophthalmologists, and rheumatologists. Prolonged and structured follow‐up is essential to monitor treatment response, detect late recurrence, and prevent irreversible visual sequelae, ultimately ensuring the best possible clinical and functional outcomes.

## Funding

No funding was received for this manuscript. Open access publishing facilitated by Universita di Pavia, as part of the Wiley ‐ CRUI‐CARE agreement.

## Disclosure

The authors have no financial or personal relationships with individuals or entities that could inappropriately influence or bias the content presented.

## Ethics Statement

This case report was conducted in compliance with institutional ethical standards and the Declaration of Helsinki. Ethical approval was not required for this single‐patient case report as per the policies of the Fondazione IRCCS Policlinico San Matteo di Pavia, provided that patient confidentiality is maintained. Given the retrospective and descriptive nature of the report, no additional interventions beyond routine clinical care were performed. The patient was evaluated, treated, and followed up at IRCCS S. Matteo Hospital Foundation (https://ror.org/05w1q1c88). The case was managed in accordance with institutional clinical protocols and standards of care.

## Consent

Written informed consent was obtained from the patient for publication of this case report, including the use of clinical details, laboratory data, and deidentified images. All identifying information has been rigorously anonymized to protect patient privacy.

## Conflicts of Interest

The authors declare no conflicts of interest.

## Supporting information


**Supporting Information** Additional supporting information can be found online in the Supporting Information section. 

## Data Availability

The data that support the findings of this study are available from the corresponding author upon reasonable request.
